# Mediation Analysis to Investigate Differences in Prostate Cancer Diagnosis Stage Through Environmental Risk Factors in Louisiana

**DOI:** 10.3390/curroncol32080416

**Published:** 2025-07-24

**Authors:** Nubaira Rizvi, Randy Hamilton, Xiao-Cheng Wu, Michael D. Celestin, Tung-Sung Tseng, Qingzhao Yu

**Affiliations:** 1 Biostatistics and Data Science, School of Public Health, LSU Health—New Orleans, New Orleans, LA 70112, USA; nrizvi@lsuhsc.edu (N.R.); rhami5@lsuhsc.edu (R.H.); 2 Louisiana Tumor Registry, School of Public Health, LSU Health—New Orleans, New Orleans, LA 70112, USA; xwu@lsuhsc.edu; 3 Community Health Science & Policy, School of Public Health, LSU Health—New Orleans, New Orleans, LA 70112, USAttseng@lsuhsc.edu (T.-S.T.)

**Keywords:** prostate cancer, racial disparity, mediation analysis, environmental risks

## Abstract

Prostate Cancer (PCa) is the most commonly diagnosed cancer and the second leading cause of cancer death among men. In Louisiana (LA), Black men are disproportionately diagnosed at later stages compared to White men. This study explores environmental risk factors as potential intermediate variables linking race to cancer diagnosis stage. The Louisiana Tumor Registry data included 24,647 male patients diagnosed with PCa in LA between 2010 and 2018. Among them, 15,875 (64.40%) were Caucasian American (CA) and 8772 (35.59%) African American (AA). Mediation analysis using multiple additive regression trees (MART) identified possible intermediate variables that potentially explain the observed disparity. The study found that individual characteristics and environmental factors jointly explained 84% (95% CI: 44.1%, 94.6%) and 18.6% (95% CI: 7.3%, 53.7%) of the observed racial disparity in PCa stage at diagnosis, respectively. Individual factors included BMI (35.9%), marital status (28.5%), CDI (8.2%), female-headed households (2.3%), comorbidity (3.9%), and insurance status (6.3%). Environmental contributors included cancer risk due to air toxicity exposure (7.2%), asthma prevalence (6.6%), acetaldehyde levels (2.1%), railroad proximity (2.1%), walkability (0.3%), and ozone level (−0.1%). Environmental factors jointly played a significant role in the observed racial disparity. The factors such as air toxicity, acetaldehyde levels, and asthma prevalence highlight the need to address industrial pollutants to reduce the differences.

## Simple Summary

Prostate cancer is the most commonly diagnosed cancer in men and a leading cause of cancer deaths. In Louisiana, Black men are more likely than White men to be diagnosed at a later stage of the disease. This study analyzed data from 24,647 prostate cancer patients to explore reasons for this disparity. There is a significant (*p*-value < 0.001) association between race and stage at diagnosis. It found that both individual factors, like body mass index, marital status, health insurance, and environmental factors, such as exposure to air pollution, proximity to railroads, and asthma rates, affect the observed disparity. Harmful air pollutants like acetaldehyde and general air toxicity were among the key environmental contributors. Together, these factors help explain much of the difference in diagnosis stage between Black and White men. The findings highlight the importance of addressing exposure to environmental hazards and improving healthcare access to reduce racial differences in prostate cancer outcomes.

## 1. Introduction

Prostate Cancer (PCa) is the most common cancer diagnosed in men and the second leading cancer caused death in males [1,2,3]. In the U.S, more than 240,000 men are diagnosed with PCa per year, with a reported 28,000 male case deaths annually [4]. In the early to late 2010s, African American (AA) men have reported a greater incidence rate and more than twice the risk of mortality when compared to Caucasian American (CA) men [5]. Most of the literature concludes that biological, socio-economic, and healthcare-related factors are the focus of the contribution to this disparity between AA and other races [2,3,6].

Studies suggest that genetic variations, such as differences in androgen receptor activity and variations in genes like BRCA1 and BRCA2, may increase susceptibility to PCa in Black men [6]. Additionally, tumor biology differences, such as a higher prevalence of aggressive tumor subtypes in AA, contribute to poorer outcomes. Black men often also face lower socio-economic status, leading to reduced healthcare access, lower insurance coverage, fewer screenings, and delays in diagnosis and treatment [5,7,8,9]. The lack of culturally competent care and implicit biases within the healthcare system further hinder effective treatment and follow-up. Studies indicate that Black men are less likely to receive prostate-specific antigen (PSA) screenings and more likely to be diagnosed at an advanced stage, resulting in poorer prognoses [3].

In addition, environmental factors are known to play a role in racial disparities in cancer diagnosis. Several studies have shown how socio-economic lifestyle factors, including diet, physical activity, and the prevalence of comorbid conditions such as hypertension and diabetes, differ between AA and CA men and influence PCa diagnosis stage [10,11,12]. Monthly exposures to particulate matter with aerodynamic diameter ≤ 10 μm (PM10) and nitrogen oxides (NOx) have been linked with prostate cancer [13]. Another study showed greenness has a limited impact on racial disparity in prostate cancer mortality [14]. But there remain gaps in understanding how environmental risk factors contribute to the relationship between race and PCa stage at diagnosis. One study examined the link between environmental quality and prostate cancer stage at diagnosis, but it lacked investigation about how they both associate with race [15].

In Louisiana (LA), PCa remains a significant public health issue, with Black men often diagnosed at later stages compared to White men. In particular, Black residents in Louisiana’s “Cancer Alley”, bear the brunt of toxic pollution from the petrochemical industry [16]. The region along the Mississippi River, stretching from Baton Rouge to New Orleans, Louisiana, is often called “Cancer Alley” [17,18]. Also known as “Petrochemical America”, this area is home to over 150 chemical plants that refine crude oil into various petrochemicals. In Cancer Alley, an estimated 46 individuals per one million face a risk of developing cancer, significantly higher than the national average of approximately 30 individuals per one million [17]. These facilities are commonly situated on sites that were once plantations and primarily consisted of Black residents, including emancipated settlers [19,20]. The St. James Parish, Louisiana, which hosts over 100 chemical refining plants along an 85-mile stretch of the Mississippi River, has the highest percentage of African Americans [21]. Meanwhile, the Ascension parish covers 2.5 square miles on the river’s west bank, where AAs make up approximately 70 percent of the population [22]. Cancer risk of air toxins is especially high in these racially segregated areas [20,23].

While federal environmental regulations exist, such as the Clean Air Act, they have failed to protect the vulnerable communities living in these areas [24]. This has raised persistent concerns about environmental injustice in Louisiana. Black communities here are exposed to higher levels of environmental hazards [25]. This, in turn, might have contributed to Black men getting diagnosed at later stages of PCa. There remains a research gap in understanding how environmental factors affect the stage of PCa diagnosis. The systematic reviews suggest using mediation analysis to properly understand the underlying mechanism of racial disparity in PCa [26]. This research aimed to identify potential environmental factors and estimate their effect sizes that can explain the racial disparity observed in PCa stage at diagnosis using a novel multiple mediation analysis method in Louisiana.

## 2. Measurements and Methods

### 2.1. Study Population

The study was based on data collected by the Louisiana Tumor Registry (LTR) as part of the National Cancer Institute (NCI)-funded Surveillance, Epidemiology, and End Results (SEER) Program from 2010 to 2018 [27]. The LTR linked the information on prostate cancer and related variables with 2010 U.S. census tract-level residential risk factors data collected for U.S. census tracts from National Scale Air Toxics Assessment (NATA) and with environmental justice indicators [28]. The Environmental Justice Index (EJI) is a nationwide place-based tool developed by the Centers for Disease Control and Prevention (CDC) and the Agency for Toxic Substances and Disease Registry (ATSDR) [29]. It was designed to assess cumulative environmental burdens and their impacts on health, with an emphasis on advancing environmental justice and health equity [29]. During this period, 25,713 AA and CA patients over the age of 18 were identified with prostate cancer (ICD-10-CM Code C61), a primary cancer type in Louisiana. Among these, stage at diagnosis was available for 24,647 patients with 8772 (35.59%) AA and 15,875 (64.41%) CA men. Patients with an unknown diagnosis stage and race were excluded from the analysis.

### 2.2. Variables

#### 2.2.1. Predictor Variable

Race was considered the primary predictive variable, and there were two categories: African American (AA) and Caucasian American (CA), either Hispanic or non-Hispanic. CA is used as the reference group.

#### 2.2.2. Intermediate Variables

Initially, 168 variables regarding patients and their environment were considered independent variables. Patient-related variables included demographic and socio-economic factors. While environment-related variables consisted of environmental pollutants such as dichloromethane, toluene, acetaldehyde, diesel particulate matter, particulate matter 2.5, etc., from U.S. census tract level data and the EJI’s social vulnerability module, environmental burden, and health vulnerability measures. The environment burden module included variables ranging from air pollution and proximity to potentially hazardous and toxic sites to transportation infrastructure. The detailed list of the EJI’s indicators can be found at [29,30,31], and air toxins assessed by NATA are reported in [32]. EJI’s indicators involved estimates, percentile ranks for those estimates, and summed indicators or overall scores [31]. Only 12 variables were significantly associated with both race and stage at PCa diagnosis and were incorporated in the mediation analysis model. The patient attributes included were marital status (married vs. not married), insurance (private vs. public or no insurance), comorbidity (0 vs. 1–2 vs. 3 or above), and body mass index (BMI). The census tract-level variables included communities disproportionately impacted (CDI), the percent of female-headed households (Female HH), and total acetaldehyde level (AcetTot). Environmental factors included air pollution-related indices such as the probability of contracting cancer throughout a lifetime, assuming continuous exposure to air toxicity (EPL_totcr) [29,30,31]. Air toxics cancer risk is a composite metric that evaluates the potential cancer risk from inhaling 140 hazardous air pollutants (HAPs) [29]. In addition, an estimate of the mean annual percentage of days exceeding the 8 h ozone regulatory standard averaged over three years (E_ozone) is considered. Factors related to the built environment included the estimate of block groups according to their relative walkability (E_wlkind), where the walkability index represents the lack of walkability at the census block level, and the estimate of proportion of a tract’s area within 1 mi buffer of railroad (E_rail), which is a significant source of noise pollution. Lastly, the health vulnerability module included the percentile ranks of the percentage of individuals with asthma (EP_asthma), representing asthma prevalence among adults, greater than 66.66% of U.S. census tracts.

#### 2.2.3. Outcome Variable

For stage at diagnosis as outcome variable, the American Joint Committee on Cancer (AJCC) stage was dichotomized as “early stage” if stage at diagnosis was stage I or II, and “late stage” if otherwise.

### 2.3. Statistical Analysis

#### 2.3.1. Variable Selection

Descriptive analysis of all possible factors and their association with both race and stage at diagnosis was performed using the chi-squared test for categorical variables or the ANOVA test for quantitative variables. We set a high significance level of 0.10 to include potential intermediate variables. A total of 44 variables were found to be related to both race and stage out of 168 variables. A correlation test was performed between the associated variables, and only one variable was kept from each pair with a significantly high correlation (>0.8) to reduce multicollinearity. These remaining 33 variables were further screened using the data.org() function in the “mma” package in R (version 4.5.0) to identify potential intermediate variables [33]. A total of 12 variables were selected as potential intermediate variables. We also considered two joint effects of these variables. The first joint effect consisted of the 6 individual related variables—marital status, insurance, BMI, comorbidity, CDI, and percent of female-headed households. The second joint effect was a group of 6 environment-related variables—acetaldehyde, ozone, walkability, asthma, railroad, and air-toxicity exposure. Table 1 includes the descriptive statistics of the 12 variables significantly associated with both and included in the model.

#### 2.3.2. Mediation Analysis

**Figure 1:**
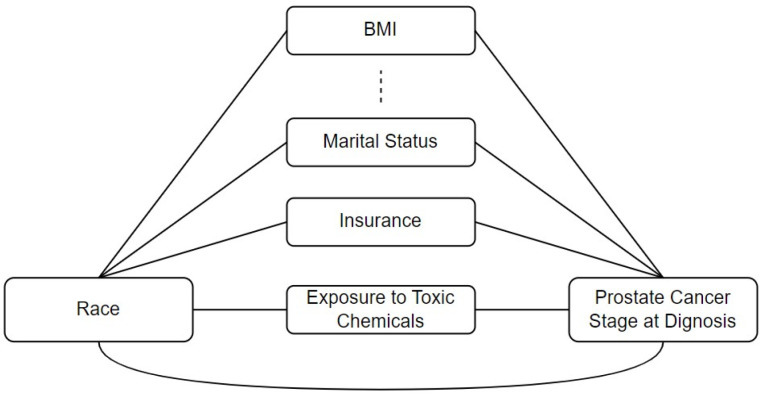
The conceptual model depicts the relationship between race and prostate cancer stage at diagnosis through potential intermediate variables.

When examining the relationship between a predictor (X, e.g., race) and a response variable (Y, e.g., the stage at prostate cancer diagnosis), multiple intermediary variables may exist within the pathways connecting them. These intermediary variables, often referred to as mediators or confounders, are associated with the predictor and are risk factors for the outcome. Utilizing multiple mediation analyses, we can decompose the association to identify the indirect effects mediated through each intermediary variable [34]. The residual effect between the predictor and the outcome, which is not accounted for by the intermediary variables, is termed the direct effect. The direct effect is the racial disparity that cannot be explained by all the intermediate variables included in the model. Figure 1 illustrates the conceptual model used to investigate racial disparities in prostate cancer diagnosis stages.

The traditional mediation approach by Baron and Kenny uses multiple regression models to assess whether a mediator carries the effect of an independent variable to the outcome variable [35]. However, it cannot be used directly if the relationships between the variables are not linear. In such cases, the counterfactual framework can be used [36]. This approach assesses mediation effects by comparing the potential outcomes corresponding to two different values of the intermediate variable. We used the method detailed by Yu et al. in [34,37] to decompose the total effect (TE) into indirect effect (IE) and direct effect (DE). This method is a generalization based on the traditional counterfactual framework. It relies on the following four assumptions.

A1: There are no unmeasured factors that confound the relationship between exposure X and outcome Y.
A2: There are no unmeasured factors that confound the relationship between exposure X and intermediate variable M.
A3: There are no unmeasured factors that could confound the relationship between intermediate variable M and outcome Y.
A4: Any intermediate variable M_i_ does not precede other intermediate variables M-_i_ causally, where M-_i_ is the vector of intermediate variables M without M_i_.

The average total effect (TE) is defined as the average change rate of outcome Y with respect to the treatment or exposure variable, X [34,37]. When the exposure variable X has two groups (like 0 and 1), the total effect is the difference in the average outcome (Y) between those two groups. It can be calculated by finding the means of Y for each group (X = 1 and X = 0) separately, then subtracting one from the other.

The average direct effect not from M_i_ is calculated as the TE by fixing M_i_ at its marginal distribution. It involves separating the data based on X values, fitting a predictive model, and then simulating the effect of X by resampling the mediator and other variables. For a binary outcome, both the parametric method with a logistic model and the nonparametric method, using the multivariate additive regression trees (MARTs), can be used. The average indirect effect (IE) from M_i_ is calculated as the difference between the average TE and the average DE not from M_i_ [34,37].

Lastly, the relative indirect effects (RIEs) are defined as the indirect effect (IE) divided by the total effect (TE). The relative effect of M_i_ represents the proportion of the total effect that would change if M_i_ could be set to its marginal distribution while varying the exposure X. Relative effects can be negative. Thus, if we change the exposure X and the effect of a mediator (Mj) follows its marginal distribution, the overall effect of X could become bigger instead of smaller. So, instead of helping to explain the effect, it increases the magnitude of the total effect.

The multiple mediation analysis was conducted with the non-linear multiple additive regression tree (MART) model to obtain the estimates of effect sizes and variances of the estimates. The relationship between the predictor and each intermediate variable was s fitted using natural cubic splines with default values in the function for hyperparameters. A variable had to meet two criteria to be considered an intermediate variable. Firstly, it must be significantly related to the exposure. Secondly, it must also be significantly associated with the outcome. When both conditions were met, the variable was included in the analysis. The data.org() function was used to identify potential mediators based on these two criteria. However, if variables were specified jointly within the function, they were included as a potential third variable regardless of whether they met these criteria and their joint effect was considered. The two joint effects, individual and environmental, were specified in the function using the argument “joinm”. A total of 2000 bootstrap samples were generated, and the quantiles of these bootstrap estimates were utilized to derive the confidence intervals.

## 3. Results

### 3.1. Descriptive Analysis Result

The Chi-square test showed a significant (*p*-value < 0.001) association between race and stage at diagnosis of PCa. The independent variables were screened using the data.org function to identify potential intermediate variables listed in Table 1.

### 3.2. Mediation Analysis Result

Based on the quantile intervals, the direct effect of race on prostate cancer stage at diagnosis was found to be insignificant, as the 95% confidence interval contains 0 (DE: −0.022, 95% CI: −0.095, 0.071). This indicates statistically that the relationship is completely explained by all the intermediate variables included in the model. The joint effect of individual characteristics accounted for 84% (95% CI: 44.1%, 94.6%) of the observed differences in PCa stage at diagnosis due to race. Similarly, the joint effect of environmental factors explained 18.6% (95% CI: 7.3%, 53.7%) of the observed racial disparity in the relationship. The individual relative indirect effect of patient characteristics included CDI (8.2%), female-headed household (2.3%), comorbidity (3.9%), BMI (35.9%), marital status (28.5%), and insurance (6.3%). In contrast, the relative indirect effects of each environmental factor were acetaldehyde levels (2.1%), E_ozone (−0.1%), E_wlkind (0.3%), EP_asthma (6.6%), E_rail (2.1%), and EPL_totcr (7.2%). Table 2 displays the effect sizes and their corresponding 95% confidence intervals.

**Figure 2:**
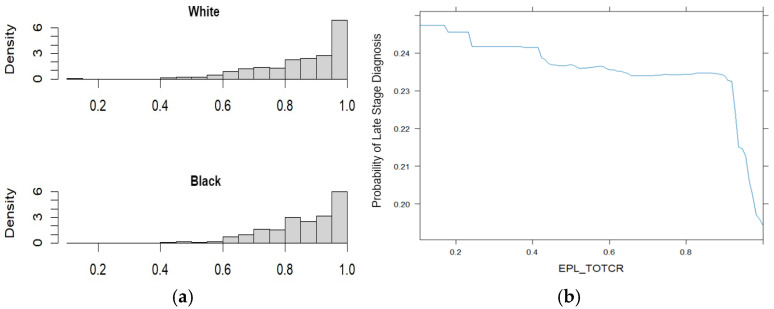
(**a**) Distribution of percentile rank of the probability of contracting cancer over the course of a lifetime, assuming continuous air toxicity exposure by race; (**b**) relationship between percentile rank of the probability of contracting cancer over the course of a lifetime, assuming continuous air toxicity exposure and the probability of late-stage diagnosis.

The package provides individual plots for each variable and a plot showing their relationship with race. Figure 2a shows the distribution of percentile rank of the probability of contracting cancer due to exposure to continuous air toxicity for White (top) and Black (bottom) individuals. Meanwhile, Figure 2b depicts the relationship between the percentile rank of the probability of contracting cancer due to exposure to continuous air toxicity and the probability of late-stage diagnosis. As the percentile rank increases, the probability of being diagnosed at a later stage decrease. More Black individuals have a higher percentile rank compared to their White counterparts, which means the chances of contracting cancer are higher for Black men from continuous air toxicity exposure. In this case, a higher likelihood of cancer risk results in earlier diagnosis.

The Supplementary Figures S1–S11 display the individual plots for the rest of the 11 variables. There was a higher proportion of White men than Black men who were married, had private insurance, had lower comorbidity, lived in census tract areas with fewer female-headed households, reduced total acetaldehyde levels, lower CDI, decreased lack of relative walkability, lesser percentile ranks of percentage of individuals with asthma, and a lower estimate of proportion of tract’s area within 1 mi buffer of railroad. These factors are related to the decreased probability of being diagnosed at a later stage compared to an earlier stage. Conversely, higher distributions of Black men were underweighted or overweight and lived in areas with lower ozone level estimates, which relates to a higher probability of being diagnosed at a later stage.

## 4. Discussion and Conclusions

This study aimed to highlight the significant role of environmental factors in explaining the racial differences in PCa diagnosis stage in Louisiana. Our findings suggest that both individual characteristics and environmental exposures contribute to the later-stage diagnosis of PCa among Black men, with individual factors jointly accounting for the larger portion of the observed disparity.

Among individual factors, BMI was the most substantial contributor, followed by marital status and insurance. Studies showed an association between obesity and PCa in AA men, which results in poorer health outcomes, including late-stage diagnosis [13]. Marital status and insurance are known to play a significant role in the racial disparity of stage at PCa diagnosis [38]. As married individuals generally have better access to healthcare, earlier cancer screening, and treatment options, a higher proportion of married White men explains part of the racial gap. Similarly, insurance coverage plays a significant role in the racial disparity observed in the diagnosis and treatment of prostate cancer among African American (AA) patients. Uninsured men are considerably less likely to receive treatment. With a higher proportion of AA men having public insurance or lacking coverage altogether, they are less likely to be diagnosed at an earlier stage, as they tend to delay the screening and treatment [39,40,41]. Comorbidity scores indicate pre-existing conditions present in individuals. A higher proportion of White men had lower scores than Black men. Higher comorbidity scores are associated with racial differences in prostate cancer mortality [42]. Additionally, higher CDI scores indicated Black residents were predominantly concentrated in more socioeconomically disadvantaged areas [43]. This study showed that higher CDI scores increase the probability of being diagnosed at a later stage. Female-headed households are an indicator of socio-economic status. Upper-middle-class suburban neighborhoods are associated with fewer female-headed households [44]. Higher proportion of Black men living in areas with higher female-headed households, which often have lower median incomes compared to dual-headed households, especially in the absence of equitable access to employment and wages [45]. This increases the likelihood of late-stage diagnosis.

Furthermore, the joint effect of environmental factors explained the remaining observed racial disparity. While the environmental effects were relatively smaller and some individual environmental variables did not reach statistical significance, they were still jointly significant, indicating that long-term environmental exposures can influence cancer outcomes, especially for Black men. This emphasizes the importance of environmental influences in explaining the racial gaps in PCa diagnosis. A higher proportion of AA men had a higher cancer risk from inhaling different HAPs like benzene, dioxin, formaldehyde, and ethylene oxide, which are known carcinogens [46,47,48]. Together, formaldehyde and benzene contribute nearly 60% of the total cancer-related health impacts [48]. The cancer risk measure considers 140 inhaled HAPs because of continuous exposure. Similarly, they reside in areas with elevated ground-level ozone levels exceeding the National Ambient Air Quality Standard (NAAQS), which is linked to adverse health outcomes [49,50,51]. Black men living in Louisiana, particularly along the Mississippi River or Cancer Alley, are particularly exposed to air pollution. This region is home to numerous petrochemical plants and fossil fuel industries. The high concentration of these facilities leads to elevated levels of toxic emissions, which disproportionately affect the predominantly Black communities residing there [19,20]. Interestingly, this heightened exposure to these chemicals could lead to earlier detection and diagnosis. This is possibly due to more frequent screening or earlier onset of symptoms in this region due to such severe exposure. However, it is important to note that while Black men may be diagnosed earlier, their exposure to continuous air toxics suggests they may face a higher overall risk of developing cancer, emphasizing the need for targeted interventions to address these disparities. Acetaldehyde is a known air pollution carcinogen known to cause oxidative damage [52]. More AA men also live in areas with higher acetaldehyde levels. Increased levels of acetaldehyde showed a higher probability of late-stage diagnosis. Prolonged exposure might allow the disease to progress undetected or contribute to a delay in diagnosis due to the absence of early symptoms. Additionally, they lived in areas with a high prevalence of asthma and within a 1-mile buffer of a railway, more than CA men, which is an indicator of poor air and noise quality, respectively. Both factors showed an association with late-stage diagnosis. Lack of walkability was also higher for areas where Black men reside. Walking in green spaces is known to have health benefits [53]. When neighborhoods are not walkable, residents may have fewer opportunities to engage in regular exercise, which helps with early detection of health issues. Moreover, reduced walkability can limit access to healthcare facilities and screenings, leading to delayed diagnoses.

Overall, poor environmental conditions can cause delays in cancer diagnosis and treatment due to limited access to healthcare and routine screenings. This environmental injustice exacerbates existing health disparities, making it crucial to address the pollution and the socio-economic barriers these communities face. AA men in heavily industrialized areas in Louisiana are more likely to experience advanced-stage PCa due to prolonged toxic chemical exposure, exacerbating racial health disparities. The link between chemical exposure, asthma, and cancer diagnosis highlights the importance of addressing long-term environmental exposures.

This study’s limitations include a lack of zip codes to connect with area-specific environmental factors. This may obscure localized environmental exposures and prevent precise assessment of area-specific factors like those in Cancer Alley. The study’s cross-sectional design limits the ability to establish causal relationships between exposure to environmental factors and cancer diagnosis stage. Additionally, the absence of a formal sensitivity analysis for unmeasured confounding may weaken the robustness of causal interpretations drawn from the mediation analysis. As some environmental factors were jointly significant but not individually statistically significant, they should be viewed as potential areas for further investigation rather than definitive contributors to racial disparities in prostate cancer stage at diagnosis. The generalizability of the findings may also be constrained as this research focuses only on data from Louisiana. Future studies should include biological pathways and the environment for thorough analysis. It should also incorporate additional environmental data, such as wind patterns and water pollution levels, to provide a more comprehensive understanding of environmental influences. Integrating genetic and epigenetic analyses would offer deeper insights into the biological mechanisms underlying racial disparities in prostate cancer outcomes. Furthermore, using geospatial mapping techniques will allow to more precisely evaluate the impact of proximity to Cancer Alley.

## Figures and Tables

**Table 1:** Descriptive statistics of potential intermediate variables associated with race and stage at diagnosis.

	AA	CA	*p*-Value *
	(*N* = 8772, 35.59%)	(*N* = 15,875, 64.41%)	
*N* = 24,647	*N* (% or SD)	*N* (% or SD)	
Stage at diagnosis			
Early stage	6905 (78.7%)	12,771 (80.4%)	<0.001
Late stage	1867 (21.3%)	3104 (19.6%)	
Marital Status			
Married	4593 (57.8%)	10,822 (76.7%)	<0.001
Not Married	3348 (42.2%)	3291 (23.3%)	
Insurance			
Private Insurance	2989 (35.6%)	6609 (43.8%)	<0.001
Public or No Insurance	5416 (64.4%)	8472 (56.2%)	
Comorbidity			
0	6765 (77.1%)	13,457 (84.8%)	<0.001
1 or 2	1736 (19.8%)	2204 (13.9%)	
3 or above	271 (3.1%)	214 (1.3%)	
BMI			
Mean (SD)	29.2 (6.25)	29.4 (5.44)	0.005
Female-headed Households			
Mean (SD)	22.8 (10.2)	13.5 (6.65)	<0.001
CDI			
Mean (SD)	0.494 (0.965)	−0.504 (0.622)	<0.001
Acetaldehyde			
Mean (SD)	2920 (14,900)	2050 (12,500)	<0.001
Ozone			
Mean (SD)	0.369 (0.502)	0.331 (0.462)	<0.001
Walkability			
Mean (SD)	7.74 (3.21)	6.75 (2.81)	<0.001
Proximity to Railroads			
Mean (SD)	56.6 (37.1)	33.6 (33.4)	<0.001
Asthma Prevalence			
Mean (SD)	10.7 (1.47)	9.26 (1.13)	<0.001
Cancer Risk from Air Toxicity			
Mean (SD)	0.864 (0.120)	0.858 (0.138)	<0.001

* *p*-values are calculated from the chi-sq test or *t*-test.

**Table 2:** Relative effects of the intermediate variables with 95% confidence intervals.

Variable Name	Indirect RE *	SD	95% CI	*p*-Value
Individual	0.84	0.127	(0.441, 0.946)	<0.001
Environmental	0.186	0.118	(0.073, 0.537)	0.012
BMI	0.356	0.069	(0.194, 0.459)	<0.001
Marital Status	0.285	0.066	(0.139, 0.394)	<0.001
CDI	0.082	0.106	(−0.034, 0.383)	0.116
EP Asthma	0.066	0.103	(−0.061, 0.343)	0.204
EPL Totcr	0.072	0.034	(0.010, 0.142)	0.024
Comorbidity	0.039	0.027	(0.020, 0.121)	<0.001
AcetTot	0.021	0052	(−0.033, 0.171)	0.268
Insurance	0.063	0.030	(0.002, 0.118)	0.036
E Rail	0.021	0.068	(−0.125, 0.138)	0.704
E Wlkind	0.003	0.041	(−0.079, 0.093)	0.712
E Ozone	−0.001	0.001	(−0.023, 0.019)	0.776
Female HH	0.023	0.126	(−0.461, 0.059)	0.256
Direct effect	−0.022	0.051	(−0.095, 0.071)	0.996

* RE = relative effect.

## Data Availability

The data supporting this study’s findings is restrictedly accessible by requesting it from the Louisiana Tumor Registry and will not be released without IRB approval. For inquiries regarding the data, researchers are encouraged to contact Lauren Maniscalco at lspiza@lsuhsc.edu for further details on the LTR’s data release policies.

## Supplementary Materials

The following supporting information can be downloaded at: https://www.mdpi.com/article/10.3390/curroncol32080416/s1, Figures S1–S11: Distribution of each variable by race and their relationship with the probability of diagnosis at a late stage.
