# Supplementary material for: Mediation Analysis to Investigate Differences in Prostate Cancer Diagnosis Stage Through Environmental Risk Factors in Louisiana
Source: Curr Oncol. 2025 Jul 24;32(8):416. doi: 10.3390/curroncol32080416 (PMC12384859; doi:10.3390/curroncol32080416)
Supplement: Supplementary file 1 [file curroncol-32-00416-s001.zip › curroncol-3642993-supplementary.pdf]

Supplementary Document

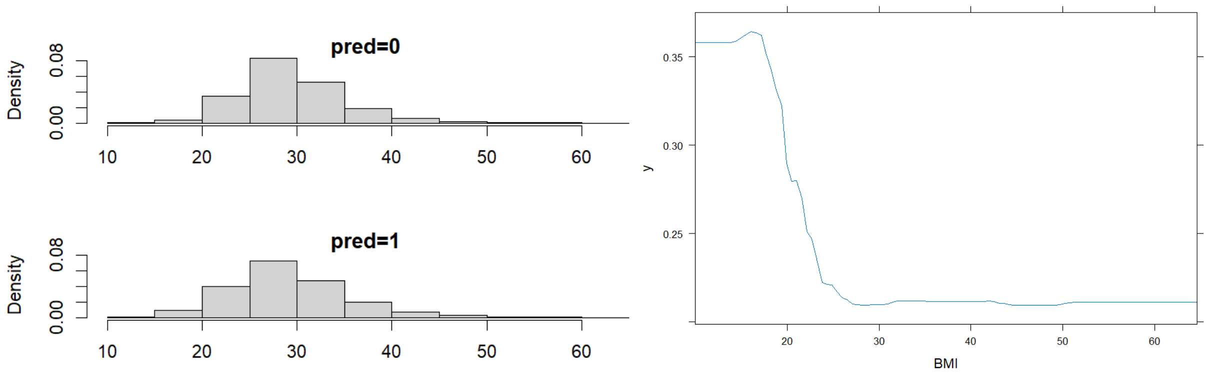

Figure S1. (**a**) Distribution of body mass index (BMI) by race; (**b**) Relationship between body mass index and the probability of late-stage diagnosis.

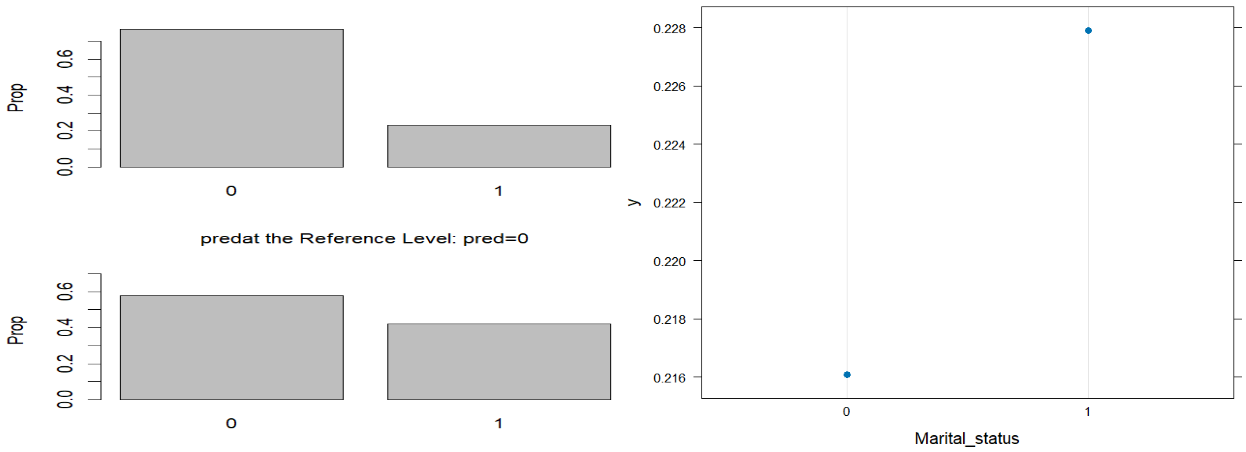

Figure S2. (**a**) Distribution of marital status (married=0 vs not married=1), by race; (**b**) Relationship between marital staus and the probability of late-stage diagnosis.

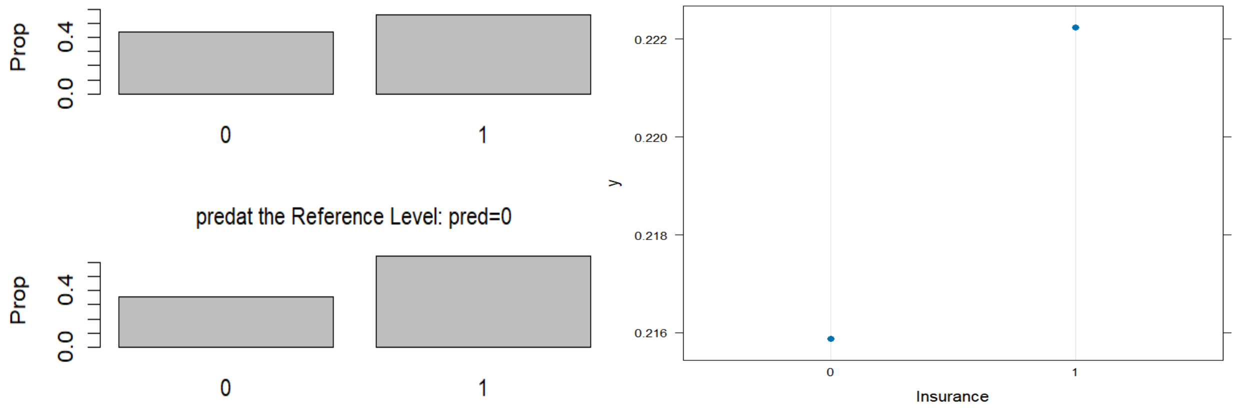

Figure S3. (**a**) Distribution of insurance (private=0 vs public or no insurance=1), by race; (**b**) Relationship between insurance and the probability of late-stage diagnosis.

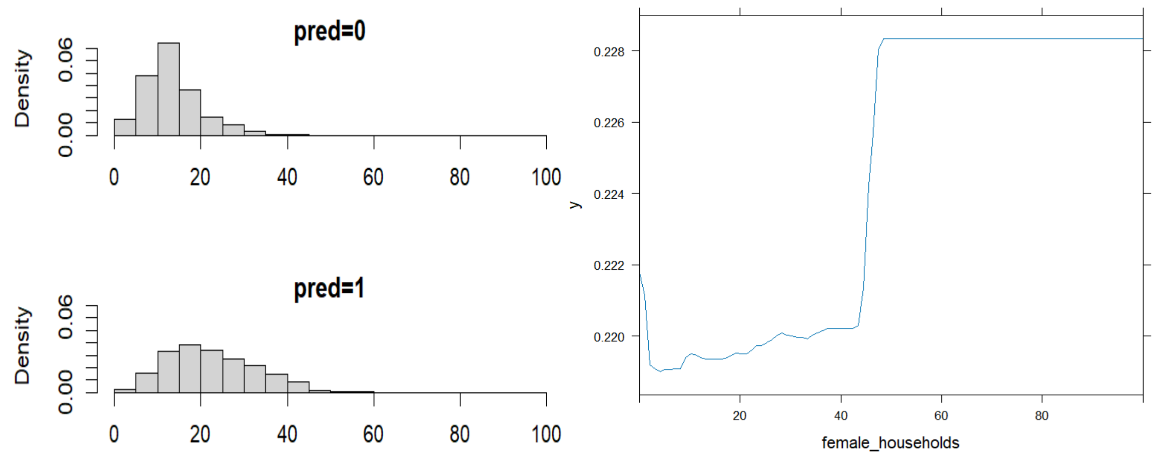

Figure S4. (**a**) Distribution of percent of female-headed households by race; (**b**) Relationship between percent of female-headed households and the probability of late-stage diagnosis.

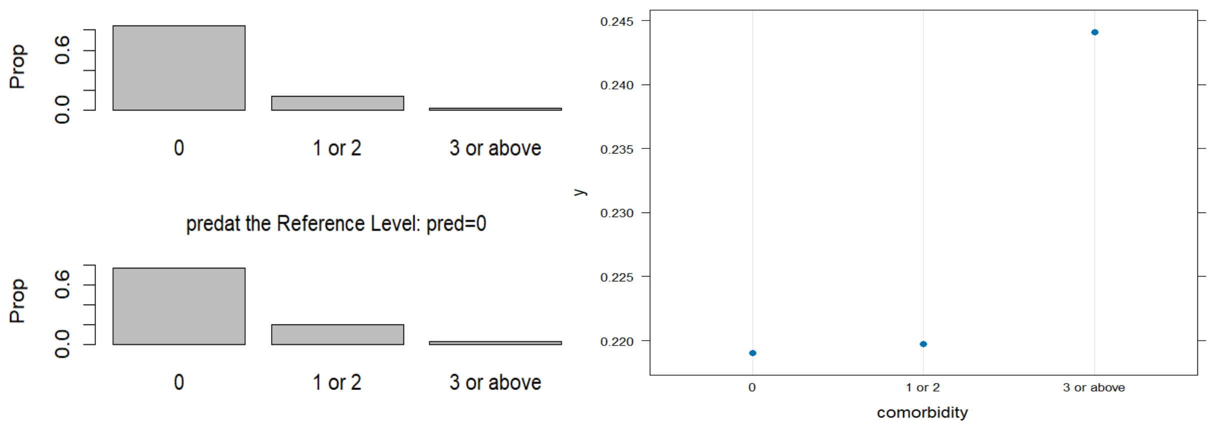

Figure S5. (**a**) Distribution of comorbidity by race; (**b**) Relationship between comorbidity and the probability of late-stage diagnosis.

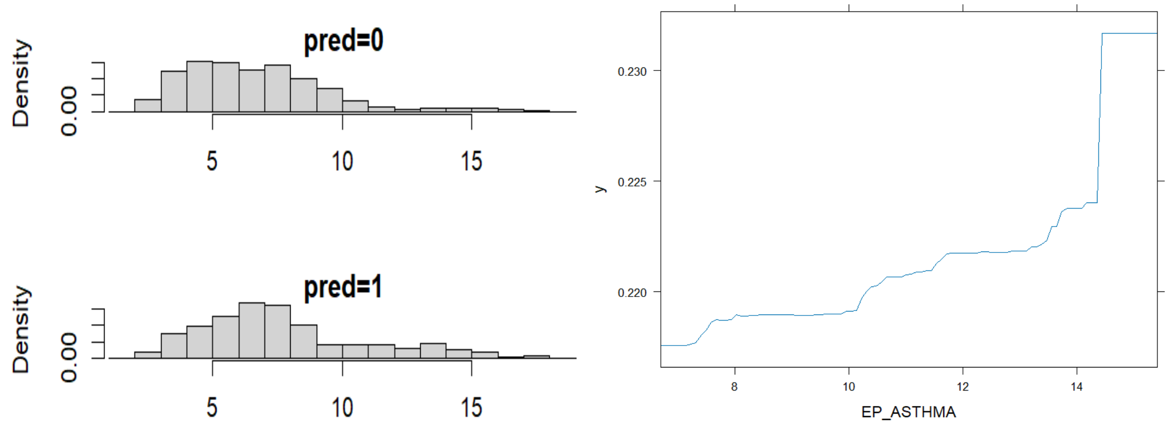

Figure S6. (**a**) Distribution of percentile ranks of percentage of individuals with asthma by race; (**b**) Relationship between percentile ranks of percentage of individuals with asthma and the probability of late-stage diagnosis.

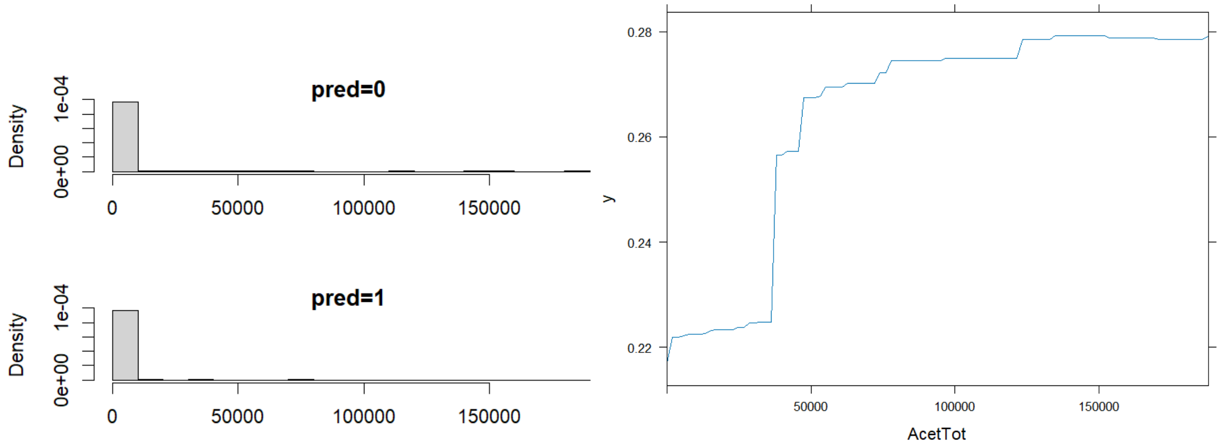

Figure S7. (**a**) Distribution of census tract area acetone level by race; (**b**) Relationship between total acetone level and the probability of late-stage diagnosis.

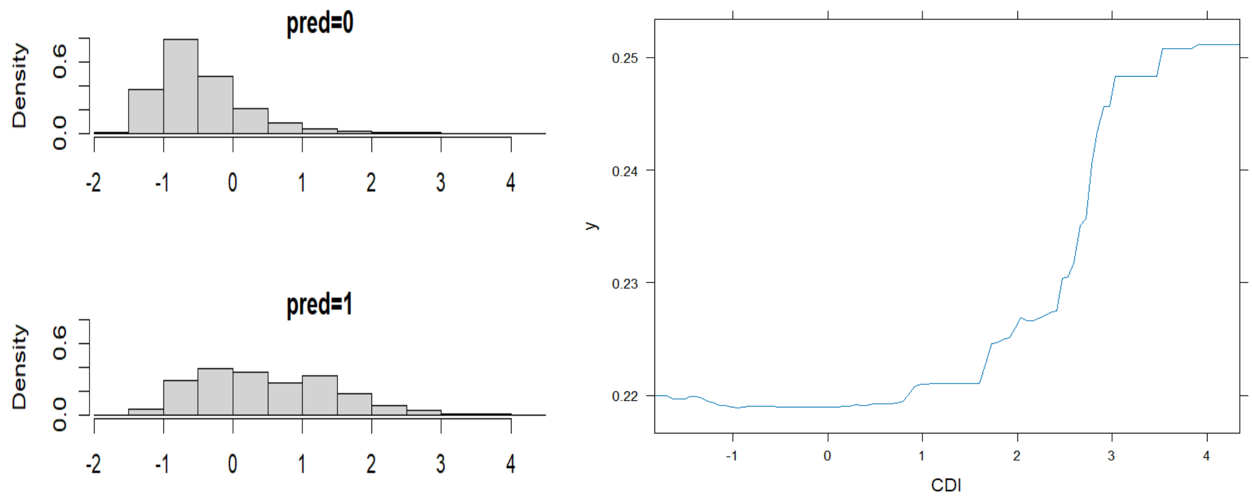

Figure S8. (**a**) Distribution of communities disproportionately impacted (CDI) by race; (**b**) Relationship between communities disproportionately impacted (CDI) and the probability of late-stage diagnosis.

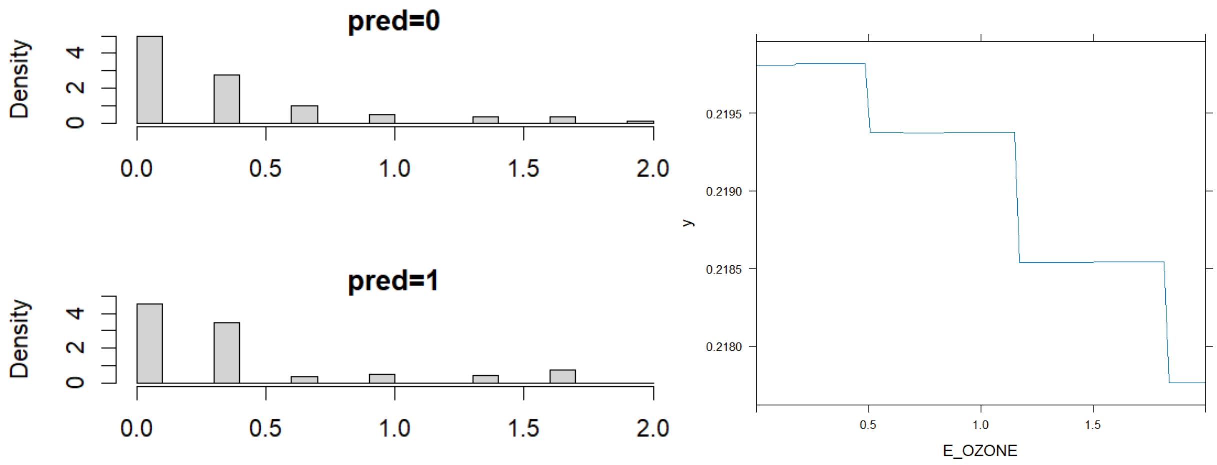

Figure S9. (**a**) Distribution of individual living in an area with estimate of 3-year average of above ozone regulatory standard by race; (**b**) Relationship between estimate of 3-year average of above ozone regulatory standard and the probability of late-stage diagnosis.

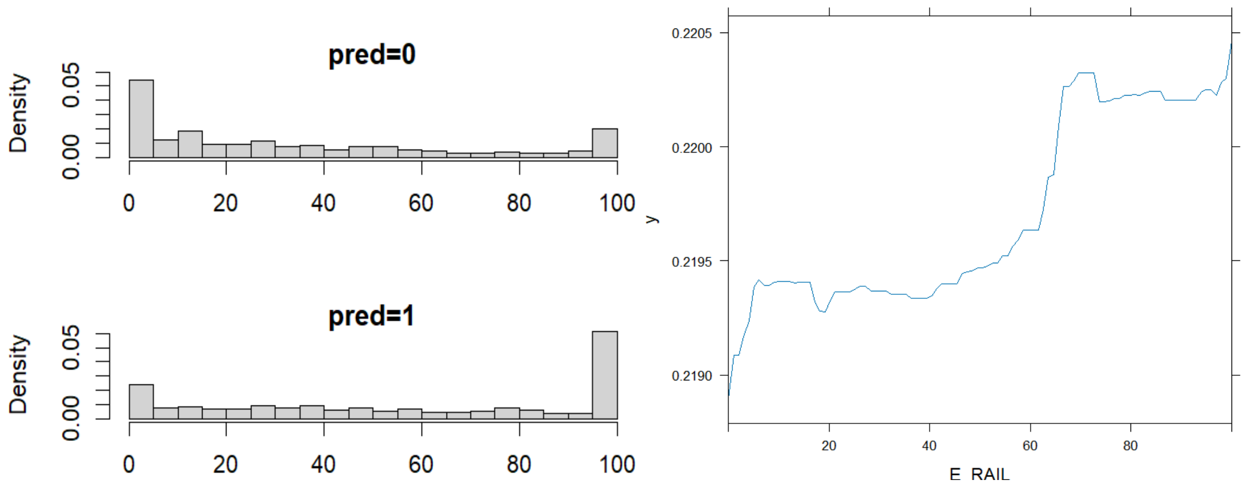

Figure S10. (**a**) Distribution of estimate of proportion of tract's area within 1-mi buffer of railroad by race; (**b**) Relationship between estimate of proportion of tract's area within 1-mi buffer of railroad and the probability of late-stage diagnosis.

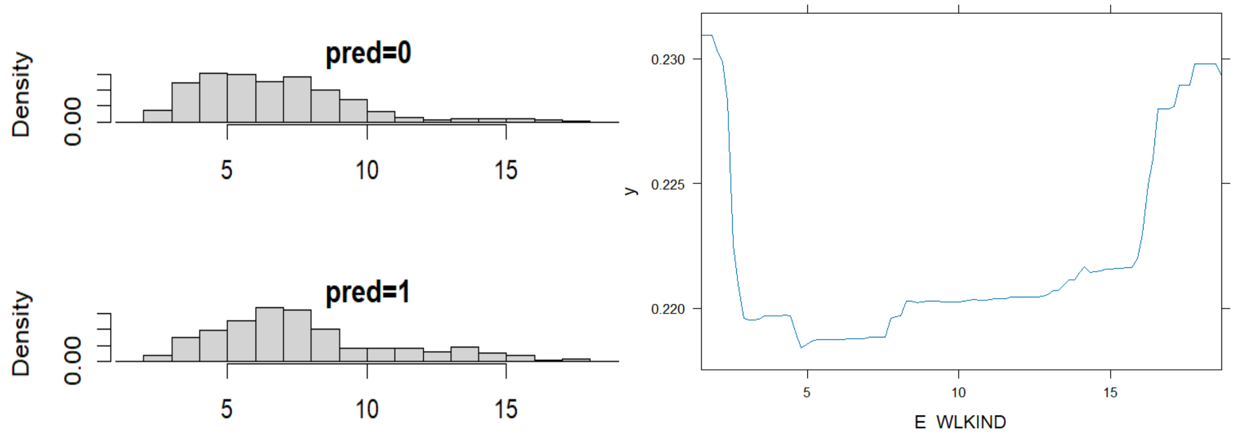

Figure S11. (**a**) Distribution of estimate of block groups according to their relative walkability by race; (**b**) Relationship between estimate of block groups according to their relative walkability and the probability of late-stage diagnosis.
